# Digitally Enabled Health Service for the Integrated Management of Hypertension: A Participatory User-Centred Design Process

**DOI:** 10.3390/ijerph182312442

**Published:** 2021-11-26

**Authors:** Vincenzo De Luca, Vanja Lazic, Strahil Birov, Klaus Piesche, Ozan Beyhan, Martino Francesco Pengo, Marcello Melgara, Marie Holm Sherman, Mikael Lilja, Antonija Balenovic, Gianfranco Parati, Maria Triassi, Raffaele Izzo, Guido Iaccarino, Maddalena Illario

**Affiliations:** 1 Dipartimento di Sanità Pubblica, Università Degli Studi di Napoli Federico II, Via S. Pansini 5, 80131 Naples, Italy; triassi@unina.it (M.T.); illario@unina.it (M.I.); 2 Dom Zdravlja Zagreb–Centar, Runjaninova ul. 4, 10000 Zagreb, Croatia; vanjlaz@gmail.com (V.L.); antonija.balenovic@dzz-centar.hr (A.B.); 3 Empirica Gesellschaft für Kommunikations-und Technologieforschung mbH, Oxfordstr. 2, 53111 Bonn, Germany; strahil.birov@empirica.com (S.B.); klaus.piesche@empirica.com (K.P.); 4 Ministry of Health of Turkey, Üniversiteler Mah. 6001. Cad. No. 9, Ankara 06800, Turkey; ozan.beyhan@saglik.gov.tr; 5 Department of Cardiovascular, Neural and Metabolic Sciences, Istituto di Ricovero e Cura a Carattere Scientifico (IRCCS), Istituto Auxologico Italiano, Via Ariosto 13, 20145 Milan, Italy; m.pengo@auxologico.it (M.F.P.); gianfranco.parati@unimib.it (G.P.); 6 Azienda Regionale per l’Innovazione e Gli Acquisti S.p.A., Direzione Generale Sanità, Regione Lombardia, Piazza Città di Lombardia 1, 20124 Milan, Italy; marcello.melgara@ext.ariaspa.it; 7 Research and Development Department, Region Jämtland Härjedalen, Kyrkgatan 12, 83127 Östersund, Sweden; marie.holm.sherman@regionjh.se; 8 Unit of Research, Education and Development, Department of Public Health and Clinical Medicine, Östersund Hospital, Umeå University, University Hospital Building 1A, 4 St., 90187 Umeå, Sweden; mikael.lilja@regionjh.se; 9 Department of Medicine and Surgery, Università Degli Studi di Milano-Bicocca, Piazza dell’Ateneo Nuovo 1, 20126 Milan, Italy; 10 Azienda Ospedaliera Universitaria Federico II, Via S. Pansini 5, 80131 Naples, Italy; rafizzo@unina.it (R.I.); guiaccar@unina.it (G.I.); 11 Dipartimento di Scienze biomediche avanzate, Università degli Studi di Napoli Federico II, Via S. Pansini 5, 80131 Naples, Italy

**Keywords:** user-centred design, digital health, hypertension, mHealth, integrated care, chronic care model, active and healthy aging, self-monitoring, health innovation, innovative procurement

## Abstract

This article describes a user-centred approach taken by a group of five procurers to set specifications for the procurement of value-based research and development services for IT-supported integrated hypertension management. The approach considered the unmet needs of patients and health systems of the involved regions. The procurers established a framework for requirements and a solution design consisting of nine building blocks, divided into three domains: service delivery, devices and integration, and health care organisation. The approach included the development of questionnaires, capturing patients’ and professionals’ views on possible system functionalities, and a template collecting information about the organisation of healthcare, professionals involved and existing IT systems at the procurers’ premises. A total of 28 patients diagnosed with hypertension and 26 professionals were interviewed. The interviewees identified 98 functional requirements, grouped in the nine building blocks. A total of nine use cases and their corresponding process models were defined by the procurers’ working group. As result, a digitally enabled integrated approach to hypertension has been designed to allow citizens to learn how to prevent the development of hypertension and lead a healthy lifestyle, and to receive comprehensive, individualised treatment in close collaboration with healthcare professionals.

## 1. Introduction

The aging population and the burden of growing non-communicable chronic diseases have increased the demand for health, social, and informal care services [1]. Demographic changes, the advent of the COVID-19 pandemic which has further increased health risks for older adults, with a consequent increase in demand for care, the increasing availability of IT solutions, and the concomitant increase in patients’ IT literacy, [2,3], and limited public human and financial resources for health and social care, are pushing health systems towards paths of digital transformation of health and care.

The European Commission (EC)’s Horizon 2020 framework program introduced Public Procurements for Innovative Solutions (PPI) and Pre-Commercial Procurements (PCP) [4,5]. These initiatives allow researchers and small and medium enterprises (SMEs) to collaborate in understanding health and care priorities and thereby allow focusing on new areas of product and solution development [6]. By promoting innovation on the demand side and orienting development towards unmet needs, innovative procurements avoid costs from unnecessary functionalities, prevent lock-in to a single vendor, and take into account the long-term needs of the public sector [7]. Addressing the needs of an ageing population translates into supporting independent living at home, self-management of age-related conditions and the reduction of isolation. Such a proactive, user-centred system requires citizens to take greater responsibility for their health and well-being, by providing information on their health status and adherence to healthy lifestyles in order to stay active and healthy as long as possible [8]. Hypertension is the most common cardiovascular disorder, affecting a third of the adult worldwide population [9]. Hypertension often occurs in the absence of symptoms, contributing to low awareness among the affected population. High blood pressure is a major risk factor for several cardiovascular diseases, such as coronary heart disease, stroke, heart failure, atrial fibrillation, and chronic kidney disease, and represents a major cause of premature death worldwide [10]. Proactive management of hypertension is key in limiting the increasing resources required to address the complications of hypertension by health and social care systems [11]. All of this requires an integrated approach to enable the more effective delivery of care and to address the complexity of the different services that patients navigate [12]. It is widely recognised that health care systems must shift from reactive disease management to health promotion and disease prevention, from a focus on disease to a focus on an individual’s wellbeing, and from fragmentation to integration of services along the continuum of care [13]. This shift can be supported by an ever-increasing volume of health data. Making this data available to patients and healthcare professionals (HCP) and the adoption of digital solutions is critical to the continuous improvement of diagnosis, treatment, and personalization of care [14,15]. Patient engagement is seen as a global concept that encompasses adherence, compliance, empowerment, health literacy, shared decision-making, and activation. Shared decision-making tools can empower patients in their disease management decisions, as well as online platforms which can support patients in communicating with their peers and health professionals, promoting self-management and health literacy [16]. Digitally supported health coaching interventions improve patients’ motivation to change their behaviours, promoting goals and reducing health risks [17]. The European Innovation Partnership on Active and Healthy Ageing (EIP on AHA) [18] has developed a Blueprint on the Digital Transformation of Health and Care in Europe [19] to facilitate the exploitation of digital solutions addressing the unmet needs of the target population, based on the “personas” user-centred approach [20].

“Pre-commercial Procurement of innovative ICT-enabled monitoring to improve health status and optimize hypertension care” (HSMonitor) is a Pre-Commercial Procurement (PCP) project aimed at leveraging state-of-the-art research and development to go far beyond current practice in the management (detection, prevention, self-management, and treatment) of hypertension. Five procurers from four EU Member States (Azienda Ospedaliera Universitaria Federico II [Italy], Dom Zdravlja Zagreb–Centar [Croatia], Lombardy Region [Italy], Region Jämtland-Härjedalen (RJH) [Sweden] and Turkish Ministry of Health [Turkey]) adopted a patient-centred approach for defining the technical and organizational specifications of each procurer. This was carried out in order to launch a Call for Tenders for research and development services to design and implement digital solutions to effectively support hypertension management.

Joint procurement pursues the goal of bringing the market for IT products closer to the unmet needs of hypertensive patients and healthcare systems by supporting business investment in research and development.

The HSMonitor procurement process is divided into a preparatory phase and three phases of competitive development.

During the preparatory phase, the Open Market Consultation was carried out through workshops organised by procurers to consult with vendors, inform them on procurers’ ambitions and set realistic and innovative procurement targets. The five procurers then launched a Call for Tenders that was published on the project website and in the EU official journal. The competitive development of the solutions is articulated in three phases:

- Phase I: Concept design, solution architecture and technical specifications;
- Phase II: Development of prototype systems;
- Phase III: Development and testing of pilot systems.

The purpose of this article is to describe the approach adopted to set up the specifications for a value-based research and development services procurement for the purpose of integrated management of hypertension. Thereby, it takes into account the related state-of-the-art on the demand and supply side of digital health market, and the unmet needs of patients and health systems of the HSMonitor regions.

## 2. Materials and Methods

eHealth and mHealth alone cannot achieve better care, but the systems must be properly integrated into the care processes, work routines, and daily lives of end users.

**Figure 1:**
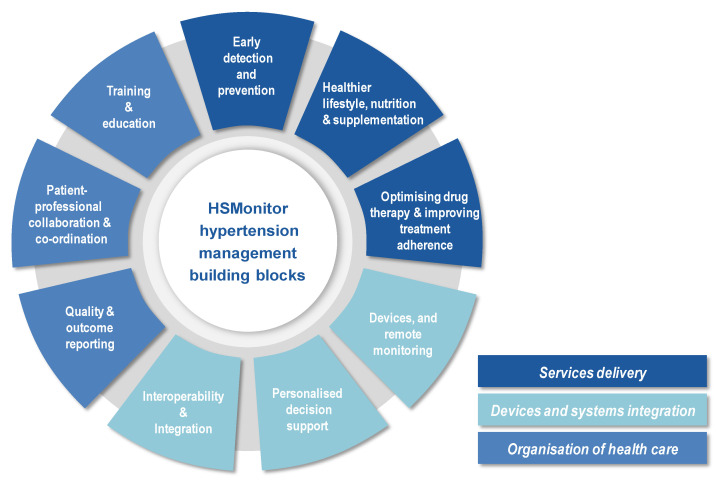
Building blocks in the HSMonitor framework.

The Chronic Care Model (CCM) asserts that improved health outcomes can be achieved with an informed, activated patient and a proactive practice team. On the part of the health system and healthcare professionals, clinical information systems and decision support tools play a key role. The patient draws on self-management support from community resources. It is the productive interactions between the two sides of the model which lead to improved health outcomes [21]. According to the desirable components for an effective chronic disease management identified by the CCM, a proactive health care team has been identified to communicate regularly with self-activated hypertension patients [22,23,24,25]. Based on this integrated management model, the HSMonitor procurers established a framework for requirements and solution design that consists of nine building blocks (Figure 1), divided into three domains: (a) service delivery, (b) devices and integration, and (c) health care organisation. The building blocks represent areas where HSMonitor procurers intend to provide significant improvements over the current state-of-the-art through innovative digital solutions.

**Figure 2:**
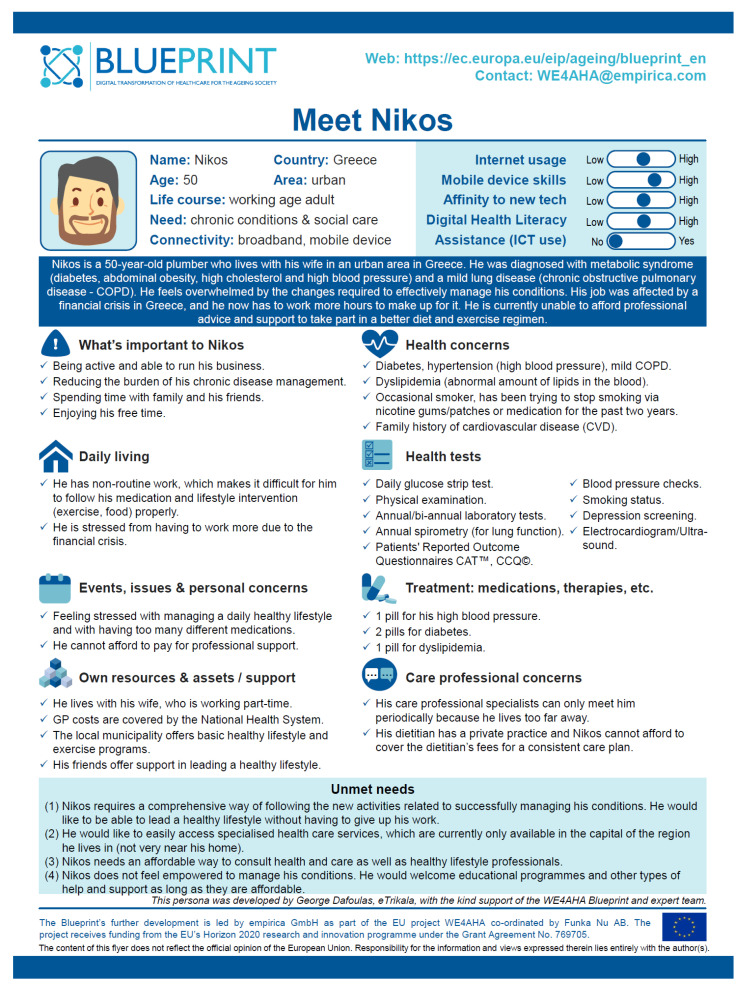
Nikos’ persona poster developed by Empirica GmbH, represents health and care needs, aspirations, attitudes, disease-related characteristics, of a chronic multimorbid patient, and identify what is important to him. Reprinted from [19].

The starting point for identifying the needs of the target population was the Nikos persona profile, which was developed as part of the European Blueprint on digital transformation of health and care [19]. Nikos is a 50-year-old Greek plumber who has been diagnosed with metabolic syndrome (diabetes, abdominal obesity, high cholesterol, and hypertension) and a chronic obstructive pulmonary disease (COPD). Nikos has a non-routine job, which makes it difficult for him to properly follow his medication and lifestyle treatment. Moreover, HCPs are only able to meet him periodically because he lives too far from the capital of his region. Nikos does not feel able to manage his condition. He is open to training programmes and other types of help and support, as long as they are accessible and fit into his work schedule (Figure 2).

To meet Nikos’ needs, HSMonitor provided a participatory design approach that runs through all phases of the PCP. The approach includes:

- The development, administration and analysis of questionnaires capturing patients’ and professionals’ views on possible system functionalities (functional requirements);
- The development of a template collecting information about existing systems at the procurers’ premises (non-functional requirements);
- The development of a set of use cases and corresponding process models, according to previously identified building blocks.

### Data Collection to Inform the Development of Functional and Non-Functional Requirements

The elicitation of technical specifications (including requirements) has been informed by multiple stakeholders which have been directly involved in the project implementation. Based on available literature, international guidelines and their experience with hypertension, or by the systems they currently have in place, the HSMonitor procurers have elaborated a first draft of the questionnaire to identify end-users’ needs and provide opinions on possible functions (functional requirements). End-users are understood as patients with (or at risk for) hypertension, health professionals who care for them, and informal caregivers who help patients with the daily management of hypertension.

The patients’ questionnaire consists of 22 multiple-choice questions and six open-ended questions to capture the creative desires of the end-users in terms of the requirements expected from HSMonitor on sharing information about the care plan, training on disease management, monitoring of clinical parameters, improving communication with HCPs, motivation and peer-to-peer support (Appendix A). The patients’ questionnaire was finalised by the project consortium and translated into Croatian, Italian, Swedish, and Turkish.

Based on the questionnaire for patients, a version for health professionals was created. The HCPs’ questionnaire consists of 15 multiple-choice questions and five open-ended questions (Appendix B). Because professionals at most procurement sites were expected to be fluent in English, the questionnaire was translated into Turkish only.

**Figure 3:**
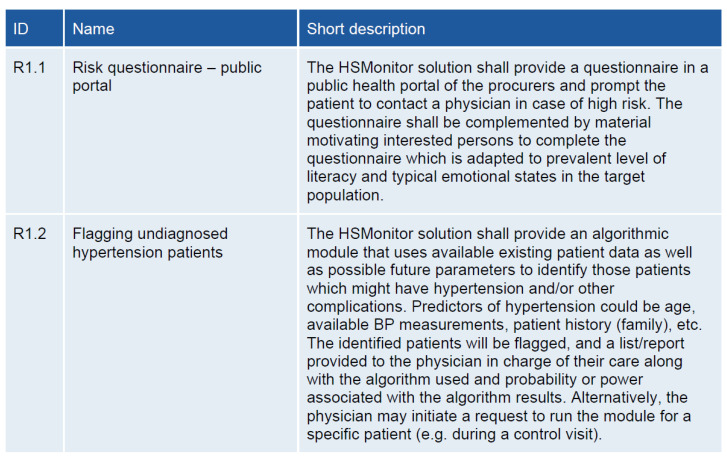
An example of HSMonitor functional requirements.

All five procurers participated in the process by appointing dedicated personnel to take part in the HSMonitor working group. Working group sessions with procurers’ stakeholders were organised to further elaborate the requirements in a joint session with all procurers, allowing the discussion of requirements and the clarification of open issues. A HSMonitor Board of Experts representing procurement, clinical, technical, and business expertise was involved in the process, providing input and feedback during the development of the requirements. Each requirement was described using ID, name, and a short description (Figure 3).

In order to provide HSMonitor suppliers with the knowledge they need to integrate the new processes with the procurers’ existing systems, a template was developed to collect information about the organization of healthcare, professionals involved, and existing IT systems at the procurers’ premises.

Use cases (UC) have often been used in the design and development of digital solutions in order to successfully meet specific user needs [26,27]. A complete set of UCs specifies all the different ways of using an IT system, and thus defines all the required behaviours of the system, delimiting its purpose.

Working group bi-weekly conference calls were conducted over a six-month period to develop common use cases and process models.

The working group identified the primary actor or user, the other key actors (with whom the primary actor interacts), the goal, and the actions or interactions among the actors needed to achieve the goal by addressing the specific needs.

**Figure 4:**
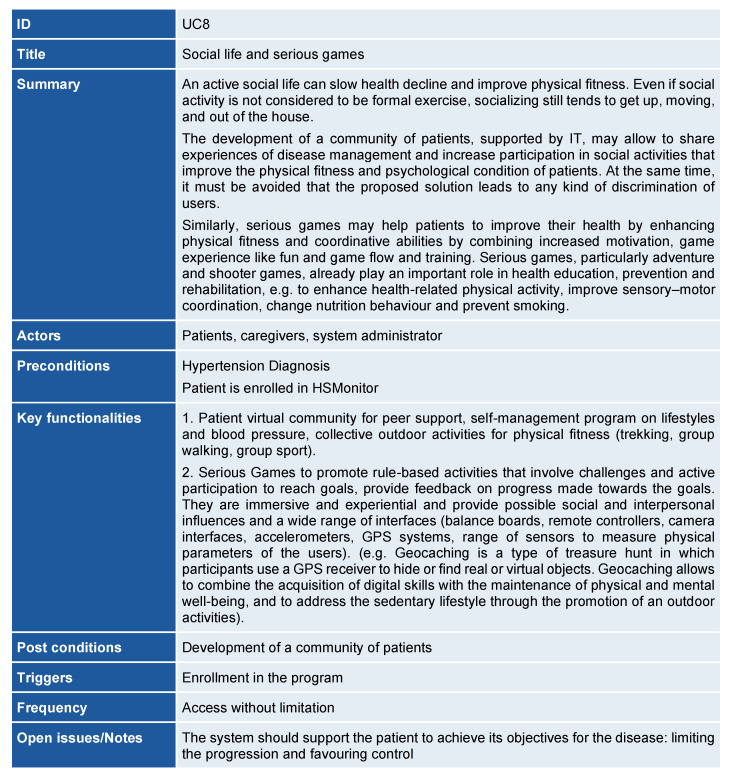
An example of HSMonitor use case.

A UC was created for each goal, maintaining the same level of abstraction. Working group members avoided over-structuring, as this can make use cases more difficult to follow (Figure 4).

In HSMonitor, each use case is described in detail with a corresponding process model with the same name and ID. The process models follow the Business Process Model and Notation (BPMN) 2.0 approach, developed by the Object Management Group (OMG) [28]. The process models aim to provide a notation that is easily understood by all supplier professionals, from the business analysts who create the initial drafts of the processes, to the technical developers responsible for implementing the technology that will run those processes, and finally, to the businesspeople who will manage and monitor those processes.

**Figure 5:**
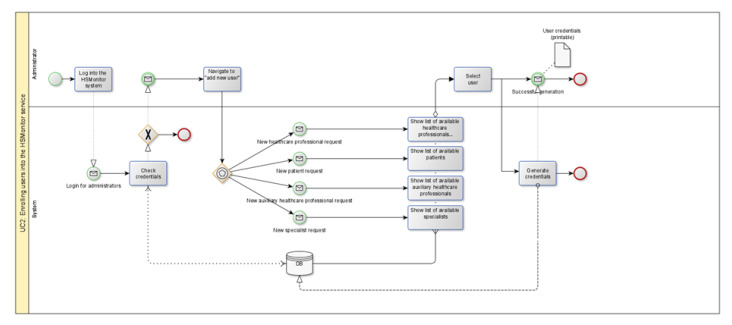
Example of HSMonitor process model.

An initial process model outline was created for each use case, which included as much detail as possible. The process models were then refined, based on feedback provided by the procurers. A final set of process models was created and validated according to BPMN technical accuracy specifications (Figure 5).

The purpose of the use cases and process model was to help suppliers better understand procurers’ expectations. Through this work, procurers initiate the dialogue planned with suppliers to adapt to their plans, request additional information from procurers, and model solutions together. UCs and process models have been continuously updated to define adjustments during the project and to document the entire process for the benefit of procurers and any other parties interested in applying the approach.

## 3. Results

### 3.1. Patients’ Questionnaire Results

The administration of the questionnaire by the five procurers took place in November and December 2019. A total of 28 patients diagnosed with hypertension representing four different age categories (18–39; 40–59; 60–79, and 80 or older) were interviewed in the five procurer regions of HSMonitor (Appendix A). The most represented was the 60–79 age group, followed by the 40–59 age group.

The majority of the patients responded that they were able to have a normal life with hypertension without major restrictions. Almost half of the participants responded that they felt optimistic about their condition and felt confident that they could live a close-to-normal life in the future. When it comes to the use of technology, most of the participants indicated that they used a mobile or smartphone with access to the internet, and more than a half used a personal computer with access to the internet.

Most patients responded that they did not have or did not know of having access to a hypertension care plan. Nonetheless, almost all stated they would like to have one.

In terms of the functionalities expected in the plan, participants would like to receive information and alerts when they should visit the doctor, setting goals in the plan (e.g., exercise goals, diet goals), reminders on how to manage and take drugs, what they can do to contribute to a good therapy or information about situations in which they should contact the doctor. In addition, patients stated that most important for them was to have online access to a shared plan, followed by being able to see the progression of the disease (e.g., systolic/diastolic blood pressure), followed by privacy and personal data protection concerns.

With regard to the training experience, only a third responded that they had previously received training focusing on hypertension management. Therefore, the participants identified topics as most relevant for a life with hypertension such as healthy diet, physical activity, medication self-management, basic health parameters that need to be constantly monitored and basic actions enabling the self-management of the disease. They also expressed their preference to receive additional training on managing them. Finally, most of the participants indicated that they would like a personalized approach with a preference towards a mixed online and Face-to-Face (F2F) training.

Regarding the measurements and health parameters the participants currently monitor, blood pressure, heart rate, body weight, blood cholesterol and sugar levels, as well as physical activity were identified as most relevant. For that purpose, most of the participants used a blood pressure monitor. Other commonly used devices are electronic scale, personal computer, and smartphone.

In relation to the communication with their physician, approximately half of the participants stated that they communicate via in person consultation, usually initiated by their physician. There was a general wish for more flexible communication.

Finally, with respect to having access to a community sharing the same condition, all participants stated that they were part of an informal community (e.g., friends’ group) but they would like to consult a dedicated hypertension community on healthy foods, physical activity and exercise, post information and exchange ideas or different parameters that need to be monitored with others.

### 3.2. Healthcare Professionals’ Questionnaire Results

The administration of the professionals’ questionnaire by the five procurers took place in November and December 2019 (Appendix B). The overall questionnaire results show that from the 26 surveyed professionals, two thirds did not have access to a care plan enabling them to co-manage their patients’ hypertension. However, they would overwhelmingly welcome the development of such a digital plan. Most of the participants found it important that such a plan should include setting individual goals together with the patient (e.g., exercise goals, diet goals), presentation of patient data according to their needs, information about what the patient can do to contribute to a good therapy, information and alerts about when the patient should visit the HCP, or if the HCP should contact them.

A third of the participants believed that a care plan should include the most relevant issues that the patient should work on together with the HCP in the following three months and thought that it should also include easy selection of information that could be printed and given together with a referral. The most important features of a hypertension care plan differ between the participants. According to them, the features of great importance are reviewing the patient’s medication and dosage; the progression of the patient’s disease; seeing the patient’s progress against the goals that have been jointly set; the online access to the plan; reliable alerts system to keep the physicians informed when necessary; the ability to update the information in the plan; sharing documents with other colleagues; and the security and protection of the personal data of the patient.

Regarding the training section, the responses reveal that only few interviewees have performed or have been present at training(s) offered to patients for managing hypertension. Additionally, 46% of the HCPs reported that the training considers the patient’s age, 38% reported that the training considers the patient’s medication, and 23% say the training ensures that patients’ personal questions are answered. HCPs reported that they would prefer to prescribe to their patients a mixed online and F2F training.

In terms of health measurements that the physicians found relevant, most of them responded that the following parameters should be recorded when treating patients diagnosed with hypertension: blood pressure, heart rate, body weight, blood cholesterol, blood sugar, physical activity, alcohol intake and tobacco use.

Based on the HCPs’ responses indicating the means and levels of communication between them and their patients, the results show that a majority of the physicians do not know if their patients adhere to the prescribed treatment. The questionnaire also reveals the most efficient methods or combination of methods for communication HCPs wish to use, namely a F2F visit initiated by the health professional, a F2F visit initiated by the patient, and a F2F visit initiated by the nurse. Only a third of them preferred phone messages, VOIP.

Regarding the community participation of the physicians, the responses show that professional community participation among professionals is low.

According to the collected answers, the most common topics professionals wish to engage in a discussion about among peers are as follows: recommendations for physical activity and exercise suitable for hypertensive patients; suitable goals doctors should set with their hypertensive patients); appropriate recommendations to hypertensive patients regarding handling stress; newest information about parameters that need to be monitored to keep the hypertension under control.

### 3.3. Functional Requirements

In total, there were 98 functional requirements (R) identified. The requirements were grouped into the nine building blocks. For each building block a rationale was given for its inclusion on the requirements list, a list of shortcomings of current care models was created and expected progress within the scope of the project was outlined. Related items were kept separated (e.g., drug-lifestyle and drug-metabolism interactions) to allow for a more precise comparison of future proposed solutions.

#### 3.3.1. Early Detection and Prevention–R1

The “Early detection and prevention” building block focused on the creation of a public portal (R1.1), implementing questionnaires to detect patients at risk (R1.2) through multiple easily accessible interfaces (R1.3). An algorithmic module that uses the acquired patient data should be able to identify the patients with hypertension. The algorithm should be accessible to both patients and care providers to use through multiple interfaces including their (Electronic Health Record)/PHR (Patient Health Record) (R1.4).

#### 3.3.2. Healthier Lifestyle, Nutrition and Supplementation–R2

R2 focused on the dietary and lifestyle modifying impact of the solution and outlined the expected features of the module. The user interaction and necessary effort to capture this data (R2.1,2,3,4)) was seen as a limiting factor, therefore, the proposed solution is expected to tackle this problem in a way that requires minimum necessary effort from the user while still being able to collect necessary data for analysis and smart nutritional recommendation (R2.5).

#### 3.3.3. Optimizing Drug Therapy and Improving Treatment Adherence–R3

R3 included the requirements for the shared care plan, by which the system will allow for the recording of medications taken according to a predetermined schedule and remind users (R3.1) of their analysis, identifying any potentially harmful drug–drug, drug-food, drug-lifestyle, and drug-metabolism interactions (R3.1,2,3,4,5).

#### 3.3.4. Devices and Remote Monitoring–R4

R4 included the devices and procedures required to obtain an appropriate set of good quality data related to the parameters associated with hypertension management, collect, and transfer them in an automatic or semi-automatic manner (R4.1,2) and visualise them in the listed mobile app platforms (R4.6). The data should be captured by two or more certified devices and sensors in a minimally obtrusive manner (R4.3,4,5).

#### 3.3.5. Personalized Decision Support–R5

R5 outlines the decision support system based on the user’s social, demographic and lifestyle parameters (non-active/office work, mobile/out-of-office work, housewife, shift work, etc.) (R5.1). It creates a framework for communication channels between patients and healthcare professionals that are informed and based on data analysis (e.g., notification in cases of deviation from goals, appropriate tips for better management, etc.)(R5.4,5,6) initiated by either party (R5.2,3) and visualized in a way that is user-friendly but contains a sufficient level of detail (R5.7) and visual guidance where suitable (R5.8).

#### 3.3.6. Interoperability and Integration–R6

R6 Outlines the interoperability requirement that includes the exchange of information (read and write data) with the systems (EHR and PHR) of all involved procurers (R6.1), the requirements on hosting (R6.2), compliance with the international interoperability standards, international code systems, international standards such as IEEE 11073 PHD for any used sensors (R6.3,4), availability of all interfaces for both desktop and mobile devices (R6.5), ease of use in different user groups (R6.6) as well as the conformity to the User Agent Accessibility Guidelines (UAAG) 2.0. at a minimum level 2 (AA) and to the Web Content Accessibility Guidelines (WCAG) 2.0 at a minimum level 2 (AA)(R6.7,8).

#### 3.3.7. Quality and Outcome Reporting–R7

R7 contains details of the necessary qualities of the reports on the data contained in the shared care plan, including, e.g., goals, alerts, medication, queries in different formats (minimum: PDF, HTML) and print-friendly, while avoiding unnecessary information and overloading users (both patients and healthcare professionals) (e.g., via a traffic light system, a sophisticated dashboard with different filters, etc.) (R7.1,2). Daily analysis and summaries of the care provided and its outcomes per patient viewed by the healthcare professional on request (pull) that include both medical (e.g., blood pressure values) and organizational (e.g., waiting times to appointment, reaction to messages sent) quality parameters should be provided and also made available for extraction (R7.3). Digital and printable user manuals covering the various interfaces and services used, available in the languages of the five procurers is also requested (R7.4).

#### 3.3.8. Patient-Professional Collaboration and Co-Ordination–R8

R8 details the properties of the shared care plan as a document used by the professional and the patient (R8.1,17) and that includes information relevant for long term hypertension care such as appointments schedule (R.2), information and visualization of the important data (R8.3,4), necessary tools for easy curating of the document from both patient user and professional user side (R5–15,18). The plan should be highly adaptable to the patient user lifestyle and set goals should be based on and influence the patients’ preferences (R8.16). The document should seamlessly exchange data with the EHR/PHR (R8.19). The shared care plan should not just act as a repository but also incorporate elements of learning (e.g., from past missed goals) and competitiveness (e.g., scoring success and being able to share it socially) (R8.20,21). Anonymised/pseudonymised data should be made available for secondary use of data for research purposes (R8.22).

#### 3.3.9. Training and Education–R9

Finally, R9 lists the requirements on education and training of healthcare personnel and users in different topics including the use of the system itself and a broad range of hypertension-related and lifestyle topics (R9–18), available in the native language of the users (R9.1).

An online platform for hypertension patients and healthcare professionals is conceptualized and its features are outlined such as the inclusion, storing and use of resources (available in community or volunteer–people- informal caregivers, images, text, links to Internet websites, etc.) (R9.19,20,21). The platform should facilitate the finding of training partners (R9.23), sharing of the data from (e.g., scores from the shared care plan)(R9.24) and creation of thematic user self-help groups (R9.25). The community platform should incorporate trust concepts to help identify users (patients or professionals) that often share good quality advice (R9.26), and rate content (R9.28). The platform should enable real-time communication between users when their status indicates availability (e.g., chat, voice message, video conferencing), especially for trusted volunteers available in the near vicinity of the user (R9.27).

### 3.4. Non-Functional Requirements

#### 3.4.1. Service Organization and Staff

To be able to define a single strategy for all procurers, a comparative analysis of the business models for managing hypertension in the HSMonitor regions was carried out. Thus, common characteristics of healthcare service organization and professionals involved were identified.

Actors involved in delivering care are:

⚬ General practitioner (GP);
⚬ Specialist (cardiologist, nephrologist, internal medicine);
⚬ Nurse;
⚬ Pharmacist;
⚬ Nutritionist;
⚬ Physical trainer.

In all the regions, GPs have the mandate of promoting prevention from the regional health system, and therefore should identify patients at risk of hypertension and assess them. Additionally, GPs are the first level of management of hypertensive patients, and therefore can request blood pressure monitoring and organ damage stratification and assessment of cardiovascular risk. Screening for hypertension is mainly performed through primary health care. Additional screening takes place on a voluntary basis during special events, such as International Hypertension Day. In Campania and Sweden, blood pressure measurements are performed in community pharmacies. All these patients are referred to the GP, who may send the patients to a second or third level clinic for further evaluation.

Specialists are consulted for selected cases when resistant hypertension or secondary forms of hypertension are suspected. Their role is also to promote and coordinate the management of GPs on their cases.

#### 3.4.2. Existing IT Systems

The procurers provided a comparative analysis of existing IT systems so that the solutions proposed by the suppliers could be interoperable with them.

In Turkey, all medical data and information coming from general practitioners, state hospitals, and university and private hospitals are collected in one data centre. The registers in the central database are analysed using business intelligence and used for clinical decision support systems. There is only one EHR portal called e-Nabız. This is fed by other information systems such as hospital information systems, telemedicine, and personal entries to the PHR side of e-Nabız.

Every public healthcare provider in Croatia is required to use the Centralni zdravstveni informatički sustav Republike Hrvatske (CEZIH), the national infrastructure for medical data storage and exchange. CEZIH provides the infrastructure for integration and interoperability and consists of applications, services, registries, and databases, includingEHR.

In the Campania region of southern Italy, there is no a centralized PHR that keeps the reports of accesses to hospital or clinic. Nevertheless, the prescription is dematerialised. The Hypertension Unit of Azienda Ospedaliera Universitaria Federico II uses the Campania Salute Network (CSN), an open registry collecting clinical information from general practitioners and community hospitals in the seven Local Health Agencies of the Campania Region.

Sistema Informativo Socio-Sanitario (SISS) of the Lombardy Region maintains a registry of all clinical documents published by the Region’s public and private health providers. The SISS is a federated information system based on the cooperation and integration of the various information systems managed independently by the healthcare facilities/organizations/participating actors. The Regional EHR is connected to a national system of interoperability, so that documents produced for non-residents are published to their own regional IT systems.

Cambio Cosmic is the medical record system for all patients in both primary and secondary care in RJH. The hospital to this has some separate IT-systems, but they are fully integrated with Cosmic. Standard e-prescriptions to the national database for pharmacies fully integrated (one-way information) in Cosmic. Laboratory results, X-ray results and all medication/prescriptions, respectively, are presented in one view for all of RJH, irrespective of where the prescription/testing was made. For other parts of the records an “own clinic” or an entire caregiver (or group of caregivers) view can be chosen. Cosmic can be used for safe communication with groups or with specific individuals, e.g., for video consultations.

### 3.5. The UCs and Process Models

**Figure 6:**
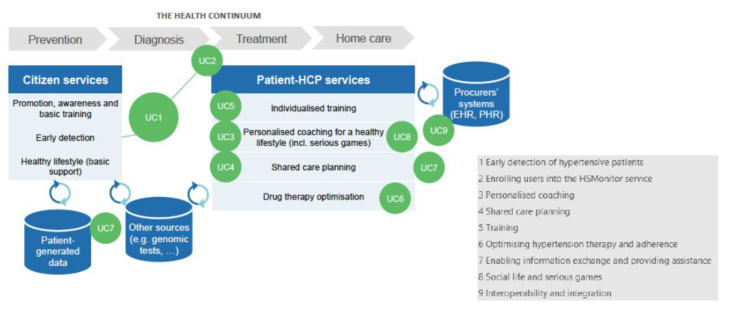
Use cases and process models covering all HSMonitor aspects.

The HSMonitor approach covers the user’s needs as they move through the continuum of care, from prevention and early detection to diagnosis, treatment, and home care (Figure 6). The patient and HCP are equal partners in the HSMonitor care planning and delivery. As a result, a digitally enabled integrated approach to hypertension has been designed that allows citizens to learn how to prevent the development of hypertension and lead a healthy lifestyle, as well as receive comprehensive, individualized treatment in close collaboration with healthcare professionals.

A total of nine use cases and their corresponding process models were defined:

⚬ UC1-Early identification of hypertensive citizens;
⚬ UC2-Enrolment of diagnosed patients and professionals in the HSMonitor service;
⚬ UC3-Personalized coaching;
⚬ UC4-Shared care planning;
⚬ UC5-Hypertension education;
⚬ UC6-Optimization of hypertension treatment and adherence;
⚬ UC7-Big data and information exchange;
⚬ UC8-Social life and serious gaming;
⚬ UC9-Interoperability and integration.

HSMonitor solutions aim to identify individuals who are at risk of developing or may already have hypertension (UC1) and to provide advice on next steps, e.g., encouraging contact with healthcare providers to confirm diagnosis (UC2).

At a technical and organizational level, the HSMonitor approach enables the transition from citizen to hypertension patient, with data generated along the pathway available to inform treatment, decisions, and goals (UC3). The digital tools support HCPs in managing all their patients through an easy-to-use shared care plan and reports (dashboards), allowing them to prioritize cases and respond more quickly to patient needs, taking into account patients’ goals, needs, and preferences (UC4). Secondary causes of hypertension, such as renal and vascular disease, hormonal diseases, medications (such as oral contraceptives (pill), corticosteroids, rheumatic medications, etc.), sleep disorders such as obstructive sleep apnoea, have been addressed by the proposed solutions.

HSMonitor solutions aim to improve the efficacy of primary prevention programs by citizens at risk for hypertension and adherence to education programs on disease management by patients (UC5).

Through more frequent data collection, the digital solutions support the HCP to identify the optimal drug therapy for a given patient. In addition to optimizing clinical parameters and medical care processes, the quality of life of affected individuals has been addressed as both a process and outcome variable (UC6).

Processing multiple data sources (e.g., from EHRs, patient-held data, data from genetic testing, data from wearables and smart scales), HSMonitor solutions provide personalized decision support, education, and coaching for improved hypertension self-management (UC7).

The IT solutions aim to share experiences of disease management among patients, both increasing participation in social activities that improve physical fitness and psychological condition, and combining increased motivation, game experience, such as fun and game flow and training (UC8). Digitally supported services are fully integrated with the healthcare organisation’s existing IT systems to enable a seamless, integrated approach to hypertension care (UC9).

## 4. Discussion

Hypertensive patients pose the challenge of a personalized therapeutic approach that requires integration with lifestyles, especially referred to adherence to prescription regimens, and physical activity. Often, such regimens are framed in the context of other diseases that complicate patient-centred care due to the need of moving through the continuum of care. The proposed approach considers patient and HCP as equal partners in the HSMonitor care planning and delivery, resulting in a digitally enabled integrated approach, engaging the citizen in leading healthy lifestyles while receiving comprehensive, individualized treatment in close collaboration with healthcare professionals. HSMonitor also provides the opportunity to target persons at risk of developing hypertension through personalized decision support, education, and coaching that are pivotal to avoid or delay disease onset and progression. Hypertension is a highly prevalent disease, and although relatively easy ease to treat, it shows heterogeneous outcomes that are influenced by educational background, ethnicity, and age that, for example, can hinder access to primary care [29]. mHealth solutions such as HSMonitor can play a crucial role in addressing some of these inequalities, providing the opportunity for remote management and telemonitoring that have already proved effective for such patients. Further efforts should be devoted to overcome the elements preventing a broader use of mHealth solutions: the approach provided by innovative procurements offers the opportunity to customize tools towards user-friendly, interoperable solutions that can also support, locally, the integration of multiple data sources (e.g., from EHRs, patient-held data, data from genetic testing, data from wearables and smart scales) and improve diagnostic-therapeutic planning in terms of outcomes, cost and quality of life for patients and quality of work for professionals.

## 5. Conclusions

This article presented an approach for designing digital health and care solutions combining User-Centred Design and Use Case Driven Approach. The combined framework allowed for a holistic representation of the unmet needs of end users (patients and professionals). The framework was developed in a collaborative approach by a dedicated team in an effort to implement human-centred design and maintain a human element throughout the process. The requirements and UCs were used to launch a call for tenders for research and development services for the development of innovative digital solutions; 17 valid tenders responded and were made available to the Evaluation Committee. The approach can also be applied to guide future user-centred development of digital solutions for the management of other diseases, particularly non-communicable chronic diseases, taking into consideration the specific needs emerging from the local contexts of adoption, the degree of maturity of the IT infrastructure, the level of IT literacy of the users, and the healthcare organizational model.

## Data Availability

The data presented in this study are available on request from the corresponding author. The data are not publicly available due to the above-mentioned article 89 of the EU General Data Protection and Regulation. The questionnaires collected information on pseudonymized patients and health professionals. The questionnaires were distributed among the centers. The electronic data was transmitted with the modern cryptography systems over the web and stored in a locked, password-protected computer.

## Appendix A. HSMonitor Patients’ Questionnaire

**Table:**

		Scores (%)
**Q1. What age group are you in?**		
18–39		1 (3.6)
40–59		11 (39.3)
60–79		15 (53.5)
80 or older		1 (3.6)
**Q2. What statement best describes your current life with hypertension?**		
I am diagnosed with hypertension, but I feel no restrictions.		18 (64.3)
I have early symptoms of complications due to hypertension, but my quality of life still is very good.		2 (7.1)
My hypertension causes minor restrictions in my life.		3 (10.7)
My daily life activities are impaired due to my hypertension or its complications.		5 (17.9)
I am not able to work because of my hypertension or its complications.		0 (0.0)
**Q3. How do you feel about your hypertension? ***		
I have hypertension, but feel confident that I can live a close-to-normal life in the future		13 (46.4)
I am worried about the negative effect hypertension may have on my life expectancy		7 (25.0)
I am worried about the reduced quality of life my hypertension may cause in the future		4 (14.3)
I feel well cared for		9 (32.1)
Other		1 (3.6)
**Q4. Please tick which communication devices you have. ***		
Landline at home		15 (53.6)
Mobile phone or smartphone with access to the internet		20 (71.4)
Mobile phone or smartphone without access to the internet		1 (3.6)
PC with access to the internet (modem, through cable operator, satellite, etc.)		16 (57.1)
PC without access to the internet		0 (0.0)
Tablet with access to the internet		7 (25.0)
**Q5. Do you have access to a hypertension plan, such as a document (online, paper) that contains relevant understandable information and can be used by your doctor and yourself to discuss your care?**		
Yes, I have access to such a plan online		4 (14.3)
Yes, I have access to a such a plan as paper document(s)		4 (14.3)
No		16 (57.1)
I do not know		4 (14.3)
**Q6. If you do not have access to such a plan, would you like to in the future?**		
Yes		18 (90.0)
No		2 (10.0)
**Q7. What kind of information would you like for such a plan to contain? ***		
Information and alerts about when I should visit my doctor		21 (75.0)
Setting lifestyle goals in the plan (e.g., exercise goals, diet goals)		16 (57.1)
Managing the drugs that I am taking and receiving reminders		16 (57.1)
Information about what I can do to contribute to a good therapy		15 (53.6)
The most relevant issues I should work on together with my doctor in the next three months		16 (57.1)
Information about situations in which I should get into contact with my doctor		15 (53.6)
Other		0 (0.0)
**Q8. What is most important to you in order to use such a plan? ***		
My personal data is secure and protected		13 (46.4)
I can access the plan online (Internet connection required)		17 (60.7)
I can update the information in the plan		7 (25.0)
I can share the document with my doctor and others (other doctors, family members)		10 (35.7)
I can understand the plan because it uses language which is easy to understand		11 (39.3)
I can see my progress against the goals that have been set together with my doctor		10 (35.7)
I can see the progression of my disease (e.g., systolic/diastolic blood pressure)		15 (53.6)
I can review my medication and dosage		11 (39.3)
Other		0 (0.0)
**Q9. Please provide up to three ideas and suggestions for what a shared hypertension document between you and your doctor should be able to do. Use sentences such as “I would like to be able to...” and “I wish there were...”. (Free text)**		
**Q10. With regard to the training that hypertension patients typically receive, please tick which topics are relevant to you at some point in your life with hypertension. ***		
I want to know which foods are good and which I should avoid in order to to stay healthy		17 (60.7)
I want to know what physical activity and exercise help me maintain a healthy life with hypertension		17 (60.7)
I want to know what goals I should set in my plan to manage my blood pressure well		9 (32.1)
I want to learn about the best ways to handle stress		10 (35.7)
I want to know what the medication I am taking is for and what to do if I forget to take it		12 (42.9)
I want to know about technology and what medical equipment I can use to help me manage my hypertension		9 (32.1)
I want to know about all the different parameters that need to be monitored to keep the hypertension in control		10 (35.7)
I want to know what the appropriate actions are, in order to manage my disease on my own		15 (53.6)
I want to know what complications can occur with hypertension patients and how to react		18 (64.3)
Other		0 (0.0)
**Q11. Have you received training for managing your hypertension yet?**		
Yes		9 (32.1)
No, I have not been offered any training by my doctor		19 (67.9)
No, I have declined to attend training offers by my doctor		0 (0.0)
**Q12. If you have attended any hypertension management training courses, how personalised were they? ***		
The training took into account my age (minimum: child, adult, elderly)		3 (33.3)
The training took into account my medication		4 (44.4)
The training was available in other languages than my own		0 (0.0)
The training ensured that my personal questions were answered		3 (33.3)
Other		3 (33.3)
**Q13. Which one of the following forms of training would you prefer to receive?**		
Online training (online materials, messages, videos)		6 (21.4)
Face-to-face training/physical presence		8 (28.6)
Mixed online and face-to-face training		14 (50.0)
**Q14. With time, the care process needs may have to be adjusted, e.g., new medication, change in diet. Which method would you prefer in order to master such changes? ***		
Face-to-face training with a doctor or nurse	Personalized 11 (39.3)	lecture-style 6 (21.4)
Paper materials relating to the change	specifically put together and printed for me 5 (17.9)	general (e.g., leaflets) 3 (10.7)
Online hypertension knowledge base (e.g., website, platform) which I use myself	which I can use to read and add content 9 (32.1)	that I can use to read content 7 (25.0)
Messages delivered on my mobile devices that are personalised for me	Personalized 14 (50.0)	General 1 (3.6)
**Q15. If you have attended hypertension management training course(s), how satisfied were you overall with them**		
very satisfied		4 (14.3)
somewhat satisfied		4 (14.3)
neither satisfied nor dissatisfied		1 (3.6)
somewhat dissatisfied		0 (0.0)
very dissatisfied		0 (0.0)
**Q16: Have you felt at some point the need to re-visit certain aspects of the training courses?**		
Yes		4 (14.3)
No		2 (7.1)
**Q17. Based on your experience with receiving training for hypertension, please provide up to three ideas and suggestions for improvement. Use sentences such as “I would like to see more...” and “I wish there were...”. (Free text)**		
**Q18. What parameters do you currently monitor or are aware of that relate to managing your hypertension? ***		
Blood pressure		25 (89.3)
Pulse frequency		23 (82.1)
Weight		16 (57.1)
Blood cholesterol		15 (53.6)
Body fat		7 (25.0)
Body muscle		0 (0.0)
Blood sugar (glucose levels)		13 (46.4)
Physical activity (steps walked, stairs climbed)		12 (42.9)
Alcohol intake		5 (17.9)
Tobacco use		5 (17.9)
Other		1 (3.6)
**Q19. Do you have and/or use any tools or devices to monitor such data or to generally inform yourself about your disease? ***	**Have**	**Use**
Blood pressure monitor	16 (57.1)	7 (25.0)
Electronic scale	5 (17.9)	6 (21.4)
Notebook/PC	8 (28.6)	3 (10.7)
Tablet	4 (14.3)	0 (0.0)
Smartphone	8 (28.6)	4 (14.3)
Other	1 (3.6)	1 (3.6)
**Q20. How do you currently communicate with your doctor regarding your disease? ***		
Via visitation, which the doctor initiates		17 (60.7)
Via visitation, which I have initiated		12 (42.9)
Via visitation, which the nurse has initiated		4 (14.3)
Via phone calls		6 (21.4)
Via phone messages (SMS, WhatsApp, etc.)		2 (7.1)
Via VoIP (e.g., Skype) and/or video conferencing		0 (0.0)
Via social media (e.g., Facebook, twitter)		0 (0.0)
Other		3 (10.7)
**Q21. Do you wish for a more flexible communication than the current situation?**		
Yes		14 (50.0)
No, I am satisfied with the current way of communication		11 (39.3)
**Q22. About what would you like to communicate with your doctor additionally? (Free text)**		
**Q23. Which method or combination of methods do you think would be the most efficient for you to communicate with your doctor in the future? ***		
Via visitation, which the doctor initiates		23 (82.1)
Via visitation, which I have initiated		11 (39.3)
Via phone calls		6 (21.4)
Via phone messages (SMS, WhatsApp, etc.)		11 (39.3)
Via VoIP (e.g., Skype) and/or video conferencing		9 (32.1)
Via social media (e.g., Facebook, twitter)		3 (10.7)
Other		2 (7.1)
**Q24. Based on your experience with communicating with your doctor, please provide up to three ideas and suggestions for improvement. Use sentences such as “I would like to...” and “I wish I could...”. (Free text)**		
**Q25. Are you currently part of a community that deals with the aspect of hypertension?**		
Yes, a community specialised in hypertension only		0 (0.0)
Yes, a community which deals with hypertension among other topics		0 (0.0)
Yes, an informal community (e.g., friends group)		0 (0.0)
No		26 (92.9)
**Q26. What topics would you consult the community about? ***		
I want to know which foods are good and which I should avoid to stay healthy		14 (50.0)
I want to know what physical activity and exercise helps me to maintain a healthy life with hypertension		11 (39.3)
I want to know what goals I should set in my plan to manage my hypertension well		13 (46.4)
I want to learn about best ways to handling stress		9 (32.1)
I want to know what the medication I am taking is for and what to do if I forget to take it		9 (32.1)
I want to know about technology and medical equipment I can use to help me manage my hypertension		9 (32.1)
I want to know about all the different parameters that need to be monitored to keep the hypertension in control		7 (25.0)
I want to post information and exchange ideas with others		12 (42.9)
I want to know what are appropriate actions to take in order to manage my disease on my own		8 (28.6)
I want to know what complications can occur with hypertension patients and how to react		9 (32.1)
Other		9 (32.1)
**Q27. What do you expect from a community around hypertension? Please provide up to three ideas and suggestions for improvement. Use sentences such as “I would like to...” and “I wish I could...”. (Free text)**		
**Q28 If you have some other ideas on what you would want to see added to the solution we will create, please write here: (Free text)**		

* multiple choice.

## Appendix B. HSMonitor Professionals Questionnaire

**Table:**

	Scores (%)	
**Q1. Do you have access to a hypertension plan, such as a document (online, paper) that contains relevant information and can be used by yourself or the patient to agree on the best care plan for them (including personalised goals)?**		
Yes, I have access to such a plan online	5 (19.2)	
Yes, I have access to a such a plan as paper document(s)	2 (7.7)	
No	17 (65.4)	
I do not know	2 (7.7)	
**Q2. If you do not have access to such a plan, would you like to in the future?**		
Yes	24 (92.3)	
No	2 (7.7)	
**Q3. What kind of information and functionality would you prefer for such a plan to contain? ***		
Information and alerts about when the patient should visit me or I should contact him/her	17 (65.4)	
Setting goals in the plan together with the patient (e.g., exercise goals, diet goals)	21 (80.8)	
Easy selection of information that can be printed and given together with a referral	7 (26.9)	
Presentation of patient data according to my needs (e.g., show all blood pressure measurements in the last six months)	19 (73.1)	
Information about what the patient can do to contribute to a good therapy	19 (73.1)	
The most relevant issues the patient should work on together with me in the next three months	9 (34.6)	
Other	1 (3.8)	
**Q4. What is most important to you in order to use such a plan? ***		
The personal data of the patient is secure and protected	4 (15.4)	
I can access the plan online (Internet connection required)	14 (53.8)	
I can update the information in the plan	10 (38.5)	
I can rely on alerts to inform me when necessary (e.g., to schedule a check-up, re-do a test, etc.)	11 (42.3)	
I can share the document with other colleagues	7 (26.9)	
I can see the patient’s progress against the goals we have set together	16 (61.5)	
I can see the progression of the patient’s disease (e.g., systolic/diastolic blood pressure)	17 (65.4)	
I can review the patient’s medication and dosage	18 (69.2)	
Other	2 (7.7)	
**Q5. Please provide up to three ideas and suggestions for what a shared hypertension document between you and your patient should be able to do. Use sentences such as “I would like to be able to...” and “I wish there were...”. (Free text)**		
**Q6. Have you performed or been present at training(s) offered to patients for managing their hypertension?**		
Yes	8 (30.8)	
No	17 (65.4)	
**Q7. If yes how personalised were the trainings? ***		
The training took into account the patient’s age (minimum: child, adult, elderly).	12 (46.2)	
The training took into account the patient’s medication.	10 (38.5)	
The training was available in more than one language	1 (3.8)	
The training ensured that patients’ personal questions are answered	6 (23.1)	
Other	0 (0.0)	
**Q8. Which one of the following forms of training would you prefer to prescribe to patients?**		
Online training (online materials, messages, videos)	4 (15.4)	
Face-to-face training/physical presence	2 (7.7)	
Mixed online and face-to-face training	20 (76.9)	
**Q9. Based on your experience with providing training for hypertension, please provide up to three ideas and suggestions for improvement. Use sentences such as “I would like to see more...” and “I wish there were...”. (Free text)**		
**Q10. What parameters do you currently record or think the system should collect to help when treating your patients’ hypertension? ***	**Currently recorded**	**Should be recorded**
Blood pressure	21 (80.8)	11 (42.3)
Pulse frequency	20 (76.9)	11 (42.3)
Weight	18 (69.2)	10 (38.5)
Blood cholesterol	18 (69.2)	12 (46.2)
Body fat	5 (19.2)	10 (38.5)
Body muscle	2 (7.7)	8 (30.8)
Blood sugar (glucose levels)	16 (61.5)	12 (46.2)
Physical activity (steps walked, stairs climbed)	7 (26.9)	15 (57.7)
Alcohol intake	7 (26.9)	11 (42.3)
Tobacco use	17 (65.4)	11 (42.3)
Other	2 (7.7)	2 (7.7)
**Q11. Do you know if your patients adhere to the prescribed treatment (i.e., take the drugs as prescribed, do physical activity?**		
Yes	8 (30.8)	
No	18 (69.2)	
**Q12. How do you currently communicate with your patients regarding their hypertension? ***		
Via visitation, which I initiate	18 (69.2)	
Via visitation, which the patient has initiated	18 (69.2)	
Via visitation, which a nurse has initiated	10 (38.5)	
Via phone calls	14 (53.8)	
Via phone messages (SMS, WhatsApp, etc.)	3 (11.5)	
Via VoIP (e.g., Skype) and/or video conferencing	0 (0.0)	
Via social media (e.g., Facebook, twitter)	0 (0.0)	
Other	6 (23.1)	
**Q13. Do you wish for a more flexible communication than the current case?**		
Yes	17 (65.4)	
No, I am satisfied with the current way of communication	7 (26.9)	
**Q14. Which method or combination of methods do you think would be most efficient for you to communicate with your patient in the future? ***		
Via visitation, which I initiate	14 (53.8)	
Via visitation, which the patient has initiated	11 (42.3)	
Via visitation, which a nurse has initiated	11 (42.3)	
Via phone calls	5 (19.2)	
Via phone messages (SMS, WhatsApp, etc.)	9 (34.6)	
Via VOIP (e.g., Skype) and/or video conferencing	9 (34.6)	
Via social media (e.g., Facebook, twitter) Other	0 (0.0) 7	
**Q15. Based on your experience with communicating with your patients, please provide up to three ideas and suggestions for improvement. Use sentences such as “I would like to...” and “I wish I could...”. (Free text)**		
**Q16. Hypertension treatment sometimes requires a combination of drugs at lower doses. However, some studies show that finding the right combination that works best for a specific patient can take time (sometimes years) as patients do not visit their doctors so often as being able to provide feedback and adjust the combination.** **From your experience, how many months on average are required to find the optimal combination of drugs for your hypertensive patient?**		
Average:	6.4 months	
**Q17. Are you currently part of a community that deals with the aspect of hypertension?**		
Yes, a community specialised in hypertension only	4 (15.4)	
Yes, a community which deals with hypertension among other topics	2 (7.7)	
Yes, an informal community (e.g., occasional meetings with other colleagues)	4 (15.4)	
No	16 (61.5)	
**Q18. What topics would you consult the community about? ***		
Dietary recommendations for hypertension patients	10 (38.5)	
Recommendations for physical activity and exercise suitable for hypertension patients	15 (57.7)	
Suitable goals doctors should set with their hypertension patients	12 (46.2)	
Appropriate recommendations to hypertension patients regarding handling stress	13 (50.0)	
Newest medicine and medicinal products for hypertension	10 (38.5)	
Newest technology and medical equipment that help patients manage their hypertension	9 (34.6)	
Newest information about parameters that need to be monitored to keep the hypertension under control	11 (42.3)	
I want to post information and exchange ideas with other colleagues	11 (42.3)	
Information about complications with hypertension patients and appropriate management	9 (34.6)	
Other	2 (7.7)	
**Q19. What do you expect from a community around hypertension? Please provide up to three ideas and suggestions for improvement. Use sentences such as “I would like to...” and “I wish I could...”. (Free text)**		
**Q20. If you have some other ideas on what you would want to see added to the solution we will create, please write here: (Free text)**		

* multiple choice.
